# Tau levels are higher in objective subtle cognitive decline but not subjective memory complaint

**DOI:** 10.1186/s13195-022-01060-1

**Published:** 2022-08-22

**Authors:** Kelsey R. Thomas, Alexandra J. Weigand, Lauren C. Edwards, Emily C. Edmonds, Katherine J. Bangen, Gema Ortiz, Kayla S. Walker, Mark W. Bondi, for the Alzheimer’s Disease Neuroimaging Initiative

**Affiliations:** 1 Research Service, VA San Diego Healthcare System, Building 13, 3350 La Jolla Village Drive (151), San Diego, CA 92161 USA; 2 Department of Psychiatry, University of California, La Jolla, San Diego, CA USA; 3 San Diego Joint Doctoral Program in Clinical Psychology, San Diego State University/University of California, San Diego, CA USA; 4 Banner Alzheimer’s Institute, Tucson, AZ USA; 5 San Diego State University, San Diego, CA USA; 6 Psychology Service, VA San Diego Healthcare System, San Diego, CA USA

**Keywords:** Subjective memory concern, Subjective memory complaints, Subtle cognitive decline, Neuropsychology, Tau PET, Biomarkers, Preclinical Alzheimer’s disease

## Abstract

**Background:**

The 2018 NIA-AA Alzheimer’s Disease (AD) Research Framework states that subtle cognitive decline in cognitively unimpaired individuals can be measured by subjective reports or evidence of objective decline on neuropsychological measures. Both subjective memory complaint (SMC) and objective subtle cognitive decline (Obj-SCD) have been shown to be associated with future cognitive decline and AD biomarkers. We examined whether there are differences in tau PET levels between (a) SMC− vs. SMC+ participants, (b) Obj-SCD− vs. Obj-SCD+ participants, and (c) participants with overlapping vs. discrepant SMC and Obj-SCD classifications.

**Methods:**

Cognitively unimpaired participants from the Alzheimer’s Disease Neuroimaging Initiative (ADNI; *n* = 236) were classified at baseline as positive or negative for SMC (SMC− *n* = 77; SMC+ *n* = 159) based on the first 12 items of the Cognitive Change Index and/or classified as positive or negative for Obj-SCD (Obj-SCD− *n* = 173; Obj-SCD+ *n* = 63) based on previously defined neuropsychological criteria. Analyses of covariance, adjusting for age, sex, APOE ε4 carrier status, and pulse pressure, examined the group differences in tau PET (AV-1451) using a composite standardized uptake variable ratio (SUVR) for regions consistent with Braak stage III/IV. The chi-squared tests examined the tau positivity rates across the groups.

**Results:**

Obj-SCD+ participants had higher tau continuous SUVR levels (*p* = .035, *η*_*p*_^2^ = .019) and higher rates of tau positivity (15.8% Obj-SCD− vs. 30.2% Obj-SCD+) than Obj-SCD− participants. Neither tau levels (*p* = .381, *η*_*p*_^2^ = .003) nor rates of tau positivity (18.2% SMC− and 20.1% SMC+) differed between the SMC groups. There was very little agreement between SMC and Obj-SCD classifications (42%; *κ* = 0.008, *p* = .862). Participants who were Obj-SCD+ without SMC had the highest tau PET levels and differed from participants who were SMC+ without Obj-SCD (*p* = .022). Tau levels in participants with both SMC and Obj-SCD did not differ from those with only Obj-SCD (*p* = .216). Tau positivity rates across the SMC-/Obj-SCD−, SMC+/Obj-SCD−, SMC−/Obj-SCD+, and SMC+/Obj-SCD+ groups were 10.5%, 18.1%, 40.0%, and 25.6%, respectively.

**Conclusion:**

Participants with Obj-SCD had a greater tau PET burden than those without Obj-SCD, but SMC was not associated with higher tau levels. The combination of SMC and Obj-SCD did not have higher tau levels than Obj-SCD alone. Findings add to the evidence that the Obj-SCD classification is associated with AD biomarkers and faster cognitive decline in ADNI participants, but further work is needed to validate this approach in more representative/diverse cohorts.

## Introduction

Research on Alzheimer’s disease (AD) has continued to move toward earlier detection, as intervention and prevention of AD prior to widespread neurodegeneration has become a primary goal [1]. Within the 2018 NIA-AA AD Research Framework, clinical stage 2 recognizes that someone can be cognitively unimpaired (CU) but experience subtle cognitive decline, in contrast to stage 1 in which someone is CU with no indication of subtle cognitive changes. According to this framework, subtle cognitive decline can be measured by the subjective report of cognitive decline or evidence of objective decline using neuropsychological measures [2]. The best method for identifying subtle cognitive decline remains unknown, as both subjective and “objective” (i.e., neuropsychological) approaches have been shown to be associated with future cognitive decline and AD biomarkers. While many studies have examined the associations of subjective cognitive decline—especially subjective memory concerns (SMC)—and objective subtle cognitive decline (Obj-SCD) with amyloid [3–7], there has been limited research examining the associations of these classifications with in vivo measures of tau, particularly tau positron emission tomography (PET).

Subjective cognitive decline, and SMC in particular, has received significant attention, particularly since Jessen and colleagues published the conceptual framework for subjective cognitive decline in 2014 [8]. Importantly, this cognitive marker has the potential to be an extremely simple and cost-effective way to identify people at risk for future AD-related declines relative to many biomarker methods. Subjective cognitive decline is defined as a self-experienced decline in cognition relative to previous cognitive functioning that is not due to an acute event, psychiatric disease, medical disorder, medication, or substance use [8]. Evidence remains mixed regarding the relationship between subjective cognitive decline and risk of progression to mild cognitive impairment (MCI)/dementia. Although several studies have demonstrated associations between subjective cognitive decline and objective cognitive performance [9–11], other studies show that additional factors such as clinic vs. community-based samples, subclinical depressive/anxiety symptoms, physical health conditions, and how subjective cognitive decline is measured can impact the degree to which an individual’s report of cognitive decline is associated with AD pathology or future progression to MCI/dementia [12–16]. The subjective decline in memory in particular may be a useful marker of future decline in objective measures of cognition [17]. Therefore, consistent with the approach used in the Alzheimer’s Disease Neuroimaging Initiative (ADNI), we specifically examined SMC as a marker of subjective cognitive decline.

Beyond subjective cognitive decline, there have been efforts to study the objective, sensitive neuropsychological measures that capture the earliest cognitive changes associated with AD pathology. Several studies have shown that subtle cognitive changes emerge much earlier than proposed by the amyloid cascade hypothesis [18], but these early changes have not been consistently captured due to the measures that are often used (e.g., cognitive screeners and clinical rating scales), which have poor sensitivity to detect very early changes [19, 20]. One approach to classifying subtle cognitive changes in the preclinical phase of AD that we have previously developed involves using sensitive neuropsychological measures and an actuarial definition that balances diagnostic sensitivity and specificity [4, 20, 21]. Objective subtle cognitive decline (Obj-SCD), not to be confused with subjective cognitive decline, uses both sensitive neuropsychological total scores and process/error scores, which capture how efficiently a neuropsychological test is performed and the types of errors that are produced. Obj-SCD status has been associated with faster progression to MCI/dementia, declines in everyday functioning, faster β-amyloid (Aβ) accumulation, faster increases in plasma p-tau181 levels, faster entorhinal cortex atrophy, and altered cerebral blood flow and functional MRI patterns [3, 4, 22–25]. However, the relationship between Obj-SCD and tau PET is unknown.

While the field continues to focus heavily on preclinical accumulation of Aβ, tau has a stronger relationship with cognitive decline than Aβ [26, 27], making it a key linking marker during this pre-MCI phase given that it is ultimately the cognitive changes and resulting functional difficulties that impact the lives of patients and families [28]. Thus, we examined the tau PET differences in (a) participants with and without SMC, (b) participants with and without Obj-SCD, and (c) participants with overlapping or discrepant SMC and Obj-SCD classifications. Given the literature that both SMC and Obj-SCD may have utility as methods to capture the pre-MCI phase along the AD clinical continuum, we hypothesized that SMC and Obj-SCD would be associated with greater tau burden relative to those without, and participants classified as both SMC and Obj-SCD would have the highest tau levels.

## Methods

### ADNI study

Data used in the preparation of this article were obtained from the Alzheimer’s Disease Neuroimaging Initiative (ADNI) database (adni.loni.usc.edu). The ADNI was launched in 2003 as a public-private partnership, led by principal investigator Michael W. Weiner, MD. The primary goal of ADNI has been to test whether serial magnetic resonance imaging, PET, other biological markers, and clinical and neuropsychological assessment can be combined to measure the progression of MCI and early AD. For up-to-date information, see www.adni-info.org.

### Participants

The enrollment criteria for ADNI have been previously described in detail [29]. ADNI was approved by the institutional review boards at each of the participating institutions. Written informed consent was obtained from all participants or authorized representatives at each site. The current study included 236 CU participants from ADNI 2 and 3 cohorts who had tau PET, Cognitive Change Index (CCI) [30], and neuropsychological data at their screening/baseline visit. ADNI 2 was the first cohort to include the CCI, which was used to define SMC.

### SMC and Obj-SCD classifications

Participants who had an ADNI diagnosis of dementia or were classified as having mild cognitive impairment [29] based on the actuarial neuropsychological criteria at their baseline visit [31–33] were excluded. Among the participants who were considered CU, SMC was determined based on a score of ≥ 16 on the first 12 items of the CCI which is the threshold used for SMC in ADNI [30, 34]. The CCI is a self-reported measure of cognitive change, and the first 12 items are focused on memory changes.

Participants were considered to have Obj-SCD based on the following actuarial neuropsychological criteria: performed > 1 SD below the age-/education-/sex-adjusted mean on (a) 1 impaired total test score in 2 different cognitive domains (memory, language, attention/executive), (b) 2 impaired neuropsychological process scores from the Rey Auditory Verbal Learning Test (AVLT), or (c) 1 impaired total test score and 1 impaired process score [3, 4, 24]. The total test scores involved six neuropsychological test scores and, specifically, included two *memory* [AVLT delayed free recall correct responses and AVLT recognition discrimination (hits minus false positives)], two *language* measures [30-item Boston Naming Test total correct or Multilingual Naming Test total correct and Animal Fluency total score], and two *attention/executive functioning* measures [Trail Making Test Parts A and B times to completion]. The three process scores for the Obj-SCD classification were calculated from the AVLT and included *total intrusion errors* (total non-target words said across all recall trials), *learning slope* [(trial 5 − trial 1)/5], and *retroactive interference* (trial 6/trial 5), all of which have been shown to differ between CU participants who remained stable and CU participants who progressed to MCI within 5 years of follow-up in ADNI [20].

### Tau PET

PET imaging using flortaucipir (AV-1451) was used to quantify tau burden. The details of data acquisition and processing of ADNI flortaucipir PET data are available at adni.loni.usc.edu. A composite region of interest (ROI) that included regions representative of Braak stage III/IV pathology representing moderately progressed tau pathology was used [35]. Earlier Braak stages risk possible unreliable PET measurement given the susceptibility of circumscribed ROIs such as the entorhinal cortex and hippocampus to partial voluming and therefore were not examined in the current study [35]. Prior work has demonstrated that CU individuals who are Aβ-positive demonstrate greater tau levels in regions well beyond the medial temporal lobe, including the parietal regions, than individuals who were considered Aβ-negative [36], which supports the use of regions beyond the entorhinal cortex and hippocampus. Standardized uptake variable ratios (SUVRs) were calculated by dividing the SUV for each ROI by the inferior cerebellar gray, and values underwent partial volume correction using the geometric transfer method [37]. Given the non-normal distribution of the tau PET variable, both transformed and non-transformed models were examined. The results did not change, and therefore, the raw tau PET SUVR continuous variable was used in the primary models. In the follow-up analyses, tau was dichotomized and positivity was defined as Braak III/IV SUVR > 1.51 [38].

### Demographics, clinical data, and covariates

Demographic data included participant age, sex, years of education, race, and ethnicity. Apolipoprotein E (APOE) positivity was defined as the presence of at least one ɛ4 allele. Vascular risk was measured using pulse pressure (systolic-diastolic blood pressure), which is a proxy measure for arterial stiffness [39]. Depressive symptoms were assessed using the Geriatric Depression Scale (GDS) score. ADNI only included participants with a baseline GDS < 6; thus, there was a limited range for this measure. The Preclinical Alzheimer Cognitive Composite (PACC) score has been shown to detect early cognitive changes associated with AD-related pathology [40] and was used to compare the general cognitive performance across the groups. The ADNI-modified PACC included the Mini-Mental State Examination, Logical Memory Delayed Recall, Digit Symbol Substitution Test, and the Delayed Word Recall from the Alzheimer’s Disease Assessment Scale–Cognitive Subscale. Each of the four component scores has a mean of 0 and a standard deviation of 1. Lower PACC scores represent lower performance. This cognitive measure was selected for the characterization of the groups since the individual measures included in the PACC do not overlap with any of the measures used for the actuarial classification of Obj-SCD.

Aβ PET was used to characterize the sample and was included as a covariate in follow-up analyses. Participants had either florbetapir (*n* = 92) or florbetaben (*n* = 143) PET scans that aligned with their tau PET scan (one participant had missing data). The details of data acquisition and processing of ADNI florbetapir PET and florbetaben PET data are available at adni.loni.usc.edu. A summary SUVR was calculated by dividing the mean uptake across 4 AD-vulnerable cortical regions (frontal, anterior/posterior cingulate, lateral parietal, and lateral temporal cortices) by whole cerebellar (white and gray matter) uptake. Greater retention of florbetapir or florbetaben is reflective of a greater cortical Aβ load. SUVR to centiloid transformations were then used to put both on a common metric [41]. Aβ positivity was defined using the established cutoffs of > 1.11 and > 1.08 for florbetapir and florbetaben, respectively [41].

### Statistical analyses

Agreement in SMC and Obj-SCD classifications was measured using a kappa statistic. Unadjusted analyses of variance and chi-squared tests were used to examine the group differences in demographic and clinical data used for descriptive purposes. For the primary analyses, analyses of covariance, adjusting for age, sex, APOE ε4 status, and pulse pressure, examined the group differences in tau PET SUVR as a continuous variable. Three models were run that compared (1) SMC− and SMC+ participants, (2) Obj-SCD− and Obj-SCD+ participants, and (3) SMC−/Obj-SCD−, SMC+/Obj-SCD−, SMC−/Obj-SCD+, and SMC+/Obj-SCD+ participants. Given the small sample size of the overlapping and discrepant groups, an alpha of .05 was used throughout. Follow-up analyses including Aβ PET (centiloids) as a covariate were conducted to determine if the results are impacted when adjusting for cortical Aβ burden. Next, participants were then classified as tau positive (T+) or negative (T−) based on tau PET Braak stage III/IV SUVR. The chi-squared models compared the proportions of T+ participants across the following groups: (1) SMC− and SMC+, (2) Obj-SCD− and Obj-SCD+, and (3) SMC−/Obj-SCD−, SMC+/Obj-SCD−, SMC−/Obj-SCD+, and SMC+/Obj-SCD+.

## Results

Of the 236 participants, 159 (67.4%) were classified as SMC+ and 77 (32.6%) were classified as SMC−, whereas 63 (27.7%) participants were classified as Obj-SCD+ and 173 (73.3%) were classified as Obj-SCD−. Demographics and clinical data by SMC and Obj-SCD groups are shown in Table 1. Briefly, relative to participants without SMC, SMC+ participants had higher depressive symptoms (despite the limited range) and lower global cognition. Notably, there were no differences in PET Aβ levels or rates of Aβ positivity. Relative to participants without Obj-SCD, Obj-SCD+ participants had lower global cognition and higher tau PET, but also did not differ in PET Aβ levels or rates of Aβ positivity. CCI score (i.e., degree of SMC) did not differ between Obj-SCD+ and Obj-SCD− participants.

**Table 1:** Baseline demographic and clinical characteristics of the SMC and Obj-SCD groups

	Total sample	SMC−	SMC+	*F* or *χ*^2^	*p*	Obj-SCD−	Obj-SCD+	*F* or *χ*^2^	*p*
*N*	236	77	159			173	63		
Age, mean (SD)	70.82 (6.24)	70.00 (5.59)	71.21 (6.52)	*F* = 1.96	0.162	70.80 (6.45)	70.88 (5.70)	*F* = 0.09	0.926
Education, mean (SD)	16.86 (2.21)	17.09 (2.10)	16.74 (2.25)	*F* = 1.30	0.256	16.81 (2.15)	16.98 (2.37)	*F* = 0.29	0.591
Female/woman, %	57.2%	59.7%	56.0%	*χ*^2^*=* 0.30	0.584	59.5%	50.8%	*χ*^2^*=* 1.44	0.230
Race, %				*χ*^2^ = 6.32	0.176			*χ*^2^*=* 2.75	0.600
American Indian/Alaska Native	0.4%	1.3%	0.0%			0.6%	0.0%		
Asian	1.7%	1.3%	1.9%			1.2%	3.2%		
Black	3.0%	1.3%	3.8%			2.3%	4.8%		
White	92.4%	96.1%	90.6%			93.1%	90.5%		
More than one	2.5%	0.0%	3.8%			2.9%	1.6%		
Ethnicity, %				*χ*^2^ = 2.98	0.225			*χ*^2^*=* 2.24	0.326
Hispanic/Latino	2.1%	0.0%	3.1%			2.9%	0.0%		
Non-Hispanic/non-Latino	97.5%	100%	96.2%			96.5%	100%		
Unknown	0.4%	0.0%	0.6%			0.6%	0.0%		
Pulse pressure, mean (SD)	55.40 (15.76)	57.09 (15.06)	54.58 (16.07)	*F* = 1.31	0.253	55.43 (15.20)	55.33 (17.35)	*F* = .002	0.968
APOE ε4 carrier, %	36.0%	29.9%	39.0%	*χ*^2^ = 1.87	0.171	37.0%	33.3%	*χ*^2^ = 0.27	0.604
GDS, mean (SD)	0.76 (1.11)	0.40 (0.67)	0.93 (1.24)	*F* = 12.24	< 0.001	0.71 (1.05)	0.89 (1.27)	*F* = 1.18	0.278
CCI, mean (SD)	19.65 (7.14)	13.56 (1.23)	22.60 (6.94)	*F* = 128.39	< 0.001	19.12 (6.63)	21.11 (8.24)	*F* = 3.65	0.057
PACC, mean (SD)	0.17 (2.82)	0.85 (2.69)	− 0.15 (2.83)	*F* = 6.67	0.010	0.75 (2.36)	− 1.40 (3.35)	*F* = 30.20	< 0.001
Tau PET, mean (SD)	1.43 (0.15)	1.44 (0.18)	1.43 (0.13)	*F* = 0.17	0.681	1.42 (0.13)	1.47 (0.19)	*F* = 4.03	0.046
Aβ PET^a^, mean (SD)	21.93 (30.46)	19.20 (28.26)	23.26 (31.49)	*F* = 0.92	0.340	21.69 (30.06)	22.58 (31.81)	*F* = 0.30	0.585
Aβ PET positivity, %	34.5%	29.9%	36.7%	*F* = 1.07	0.301	34.1%	35.5%	*χ*^2^*=* 0.04	0.844

*SMC* subjective memory complaint, *Obj-SCD* objective subtle cognitive decline, *APOE* apolipoprotein E, *GDS* Geriatric Depression Scale, *CCI* Cognitive Change Index, *PACC* Modified Preclinical Alzheimer Cognitive Composite, *Tau PET* Braak III/IV SUVR, *Aβ PET* β-amyloid cortical SUVR in centiloids, *F* statistic reported for one-way ANOVAs, *χ*^*2*^ statistic report for chi-square tests

^a^1 SMC+/Obj-SCD+ participant is missing Aβ PET data

There was very little classification agreement between SMC and Obj-SCD (*κ* = 0.008, *p* = .862). There were 57 SMC−/Obj-SCD− participants, 116 SMC+/Obj-SCD− participants, 20 SMC−/Obj-SCD+ participants, and 43 SMC+/Obj-SCD+ participants. Demographics and clinical data by SMC and Obj-SCD agreement/discrepancy groups are shown in Table 2. Participants without SMC (SMC−/Obj-SCD− and SMC−/Obj-SCD+) had the lowest level of depressive symptoms, followed by SMC+/Obj-SCD−, and the SMC+/Obj-SCD+ group had the highest level of depressive symptoms. As expected, the SMC+ groups had higher scores on the CCI than the SMC− groups, and Obj-SCD+ groups had lower global cognition scores than the Obj-SCD− groups.

**Table 2:** Baseline demographic and clinical characteristics of the discrepant/overlapping group

	SMC−/Obj-SCD−	SMC+/Obj-SCD−	SMC−/Obj-SCD+	SMC+/Obj-SCD+	*F* or *χ*^2^	*p*
*N*	57	116	20	43		
Age, mean (SD)	70.10 (5.51)	71.14 (6.86)	69.72 (5.94)	71.42 (5.56)	*F* = 0.69	0.560
Education, mean (SD)	17.16 (2.02)	16.64 (2.20)	16.90 (2.38)	17.02 (2.39)	*F* = 0.82	0.486
Female/woman, %	63.2%	57.8%	50.0%	51.2%	*χ*^2^ = 1.91	0.592
Race, %					*χ*^2^ = 11.64	0.475
American Indian/Alaska Native	1.8%	0.0%	0.0%	0.0%		
Asian	0.0%	1.7%	5.0%	2.3%		
Black	0.0%	3.4%	5.0%	4.7%		
White	98.2%	90.5%	90.0%	90.7%		
More than one	0.0%	4.3%	0.0%	2.3%		
Ethnicity, %					*χ*^2^ = 6.37	0.383
Hispanic/Latino	0.0%	4.3%	0.0%	0.0%		
Non-Hispanic/non-Latino	100.0%	94.8%	100.0%	100.0%		
Unknown	0.0%	0.9%	0.0%	0.0%		
Pulse pressure, mean (SD)	56.23 (12.80)	55.03 (16.29)	59.55 (20.38)	53.37 (15.61)	*F* = 0.77	0.512
APOE ε4 carrier, %	29.8%	40.5%	30.0%	34.9%	*χ*^2^ = 2.31	0.511
GDS, mean (SD)	0.39 (0.68)^ac^	0.87 (1.16)^d^	0.45 (0.69)^a^	1.09 (1.43)^bd^	*F* = 4.52	0.004
CCI, mean (SD)	13.47 (1.20)^ac^	21.89 (6.45)^abd^	13.80 (1.32)^ac^	24.51 (7.89)^bcd^	*F* = 45.90	< 0.001
PACC, mean (SD)	1.39 (1.99)^abc^	0.43 (2.47)^ad^	− 0.71 (3.16)^d^	− 1.72 (3.15)^cd^	*F* = 12.67	< 0.001
Tau PET, mean (SD)	1.42 (0.13)	1.42 (0.13)	1.49 (0.28)	1.45 (0.13)	*F* = 1.61	0.187
Aβ PET^e^, mean (SD)	17.01 (27.30)	24.00 (31.18)	25.47 (30.69)	21.21 (32.60)	*F =* 0.77	0.512
Aβ PET positivity, %	26.3%	37.9%	40.0%	33.3%	*F =* 2.59	0.460

*SMC* subjective memory complaint, *Obj-SCD* objective subtle cognitive decline, *APOE* apolipoprotein E, *GDS* Geriatric Depression Scale, *CCI* Cognitive Change Index, *PACC* Modified Preclinical Alzheimer Cognitive Composite, *Tau PET* Braak III/IV SUVR, *Aβ PET* β-amyloid cortical SUVR in centiloids, *F* statistic reported for one-way ANOVAs, *χ*^*2*^ statistic report for chi-square tests

^a^Significantly different from SMC+/Obj-SCD+

^b^Significantly different from SMC−/Obj-SCD+

^c^Significantly different from SMC+/Obj-SCD−

^d^Significantly different from SMC−/Obj-SCD−

^e^1 SMC+/Obj-SCD+ participant is missing Aβ PET data

**Fig. 1:**
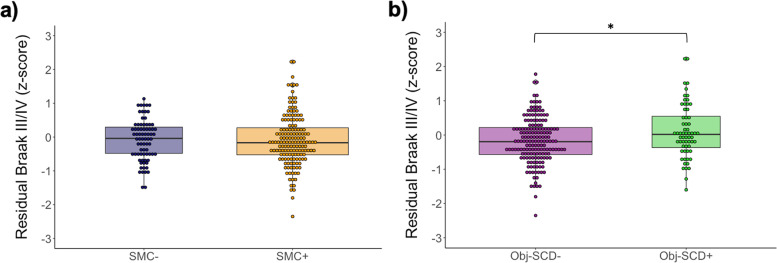
Braak III/IV SUVR by SMC status and Obj-SCD status. Dot-box plots show residual Braak III/IV values by subjective memory complaint (SMC) status (**a**) and objective subtle cognitive decline (Obj-SCD) status (**b**). Covariates included age, sex, APOE ε4 carrier status, and pulse pressure. *p < .05

When comparing continuous tau PET across the groups, after adjusting for age, sex, APOE ε4 status, and pulse pressure, there were no tau PET differences between SMC+ and SMC− participants [*F*(1, 230) = 0.77, *p* = .381, *η*_*p*_^2^ = .003; see Fig. 1]. Conversely, Obj-SCD+ participants had higher tau PET SUVRs than Obj-SCD− participants [*F*(1, 230) = 4.51, *p* = .035, *η*_*p*_^2^ = .019]. These results did not change when Aβ PET was included as an additional covariate in these models.

**Fig. 2:**
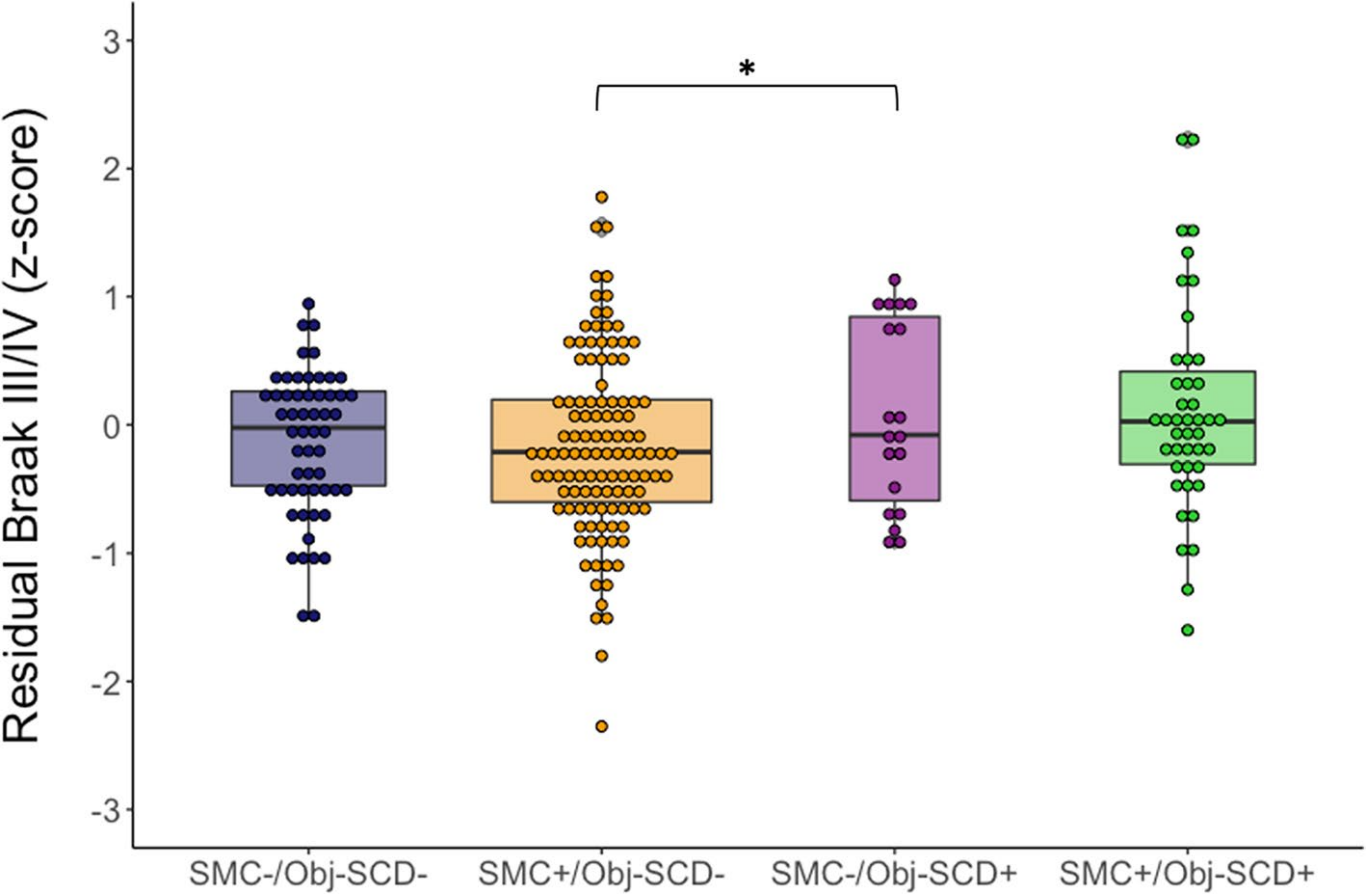
Braak III/IV SUVR by overlapping/discrepant SMC and Obj-SCD classifications. Dot-box plot shows residual Braak III/IV values by subjective memory complaint (SMC) and objective subtle cognitive decline (Obj-SCD) status. Covariates included age, sex, APOE ε4 carrier status, and pulse pressure. *p < .05

Across the four overlapping/discrepant groups, the SMC−/Obj-SCD+ had the highest tau PET SUVRs and differed from participants in the SMC+/Obj-SCD− group (*p* = .022; see Fig. 2). The SMC−/Obj-SCD+ group marginally differed from participants in the SMC−/Obj-SCD− group (*p* = .053). Tau levels in the other groups did not differ from one another (*p*-values > .213). The pattern of the results was the same when Aβ PET was included as a covariate in the models.

When participants were then classified as T+ or T−, 20.1% of SMC+ participants and 18.2% SMC− participants were T+ (*χ*^2^ = 0.13, *p* = .724). Conversely, 30.2% of Obj-SCD+ participants and 15.8% of Obj-SCD− participants were T+ (*χ*^2^ = 6.23, *p* = .013). T+ rates across the SMC−/Obj-SCD−, SMC+/Obj-SCD−, SMC−/Obj-SCD+, and SMC+/Obj-SCD+ groups were 10.5%, 18.1%, 40.0%, and 25.6%, respectively (*χ*^2^ = 9.44, *p* = .024). Relative to SMC−/Obj-SCD−, the SMC−/Obj-SCD+ (*p* = .003) and SMC+/Obj-SCD+ (*p* = .047) groups, but not the SMC+/Obj-SCD− (*p* = .197) group, had a greater proportion of T+ participants. T+ rates did not differ between SMC+/Obj-SCD+ and SMC+/Obj-SCD− (*p* = .296), nor between SMC+/Obj-SCD+ and SMC−/Obj-SCD+ (*p* = .246). SMC−/Obj-SCD+ participants had higher T+ rates than SMC+/Obj-SCD− participants (*p* = .027).

## Discussion

Our study demonstrated that participants with Obj-SCD had a greater tau PET burden than participants without Obj-SCD, with a consistent pattern of results when examining tau both continuously as a Braak stage III/IV SUVR or discretely via rate of tau positivity. Conversely, neither continuous tau levels nor rates of tau positivity differed between participants with and without SMC. Contrary to our hypothesis, the combined SMC+/Obj-SCD+ group did not evince a greater tau PET burden relative to participants with only Obj-SCD. It was instead the SMC−/Obj-SCD+ group with the highest tau PET burden, despite the small sample size. Importantly, the rate of Aβ-positive participants did not differ between the groups, and the results were not changed when Aβ PET level (in centiloids) was considered in the models.

While several studies have examined the associations of SMC or the more broadly defined subjective cognitive decline construct with Aβ PET [5, 6, 9], fewer studies have examined the associations with tau PET. One prior study demonstrated that entorhinal tau deposition was associated with greater severity of subjective cognitive decline [42]. In this study, global Aβ PET was also associated with subjective cognitive decline severity, but to a lesser degree. Within ADNI, another study examining the relationship between tau PET and subjective cognitive decline, using subscales from the Everyday Cognition (ECog) measure, showed that higher tau levels were associated with self-reported planning and visuospatial functioning, but not memory functioning [43]. Conversely, another study using ADNI data showed that the self-reported memory subscale from the ECog was associated with greater frontal tau burden, while the informant-reported memory subscale was associated with greater parietal tau burden [44]. While these studies have demonstrated some evidence of a relationship between subjective cognitive decline or SMC and tau, none of these studies examined a dichotomous subjective cognitive decline group. Although a continuous measure of subjective cognition is psychometrically advantageous, there may be less utility in the application of the results to the real world where dichotomous classifications may be particularly important for the purposes of clinical classification. Furthermore, it has been posited that the construct of subjective cognitive decline or SMC may be especially useful when screening for clinical trials [45], which would require a threshold or cut score for what is considered a meaningful report of cognitive decline. Importantly, while neither SMC nor Obj-SCD should be used alone for clinical classification or clinical trial screenings at this time [46], it is valuable to understand how these classifications overlap or differ as well as relate to AD biomarkers and future progression so that we can continue to refine these approaches and ultimately gain the information needed to empirically support clinical methods for earlier detection.

Our current study chose to use the CCI to define SMC since this is the measure that ADNI uses to define SMC and has been shown to be associated with reduced gray matter [30], worse Aβ and cerebrospinal fluid tau markers (particularly in APOE e4 carriers) [34], and faster cognitive decline relative to control participants [15]. There has been a significant effort, including the formation of the Subjective Cognitive Decline Initiative (SCD-I) working group, to define subjective cognitive decline, understand how measures of subjective cognitive decline/SMC overlap, and identify which measures are most useful [8, 17, 47, 48]. Given the many approaches to defining subjective cognitive decline/SMC, it is possible that the mixed results in the literature on SMC and AD biomarkers and future progression are due to the different approaches to defining the construct [15].

Other factors, such as the context of the study and how participants were recruited, have been shown to impact the utility of subjective cognitive decline for predicting future declines [13, 14]. Notably, despite none of the participants in this study meeting a cutoff for clinical depression levels, SMC participants had higher levels of depressive symptoms than participants without SMC. It is difficult to disentangle whether people with higher depressive or anxiety symptoms are more likely to report SMC or whether these psychiatric symptoms and SMC emerge at a similar time due to very early AD-related changes. While the latter is possible, in this current study, participants with SMC did not have higher levels of Aβ or tau relative to those without SMC. It is possible that SMC is particularly inclusive and captures a heterogeneous group of both those with and without elevated AD biomarkers or risk for accelerated cognitive declines. Importantly, we are not proposing that the concept of SMC or subjective cognitive decline should be ignored. It should remain a key consideration in evaluations of older adults as it has been associated with faster declines in some samples [7, 13] and is consistently associated with lower quality of life [49], which is particularly deserving of attention.

To our knowledge, ours is the first study to examine tau PET in participants with Obj-SCD defined using sensitive neuropsychological scores. However, the results of our study are in line with prior work showing a 4-year trajectory of medial temporal lobe atrophy [3] and accelerated increases in plasma p-tau181 over time [24]. We had previously speculated that tau deposition was likely to precede neurodegeneration and was associated with the early cognitive changes observed in Obj-SCD [3]. While longitudinal tau PET studies are needed, the current study supports this hypothesis given that there were already mean differences in Braak III/IV tau levels between participants with and without Obj-SCD at their baseline visit. Furthermore, since there were no Aβ PET differences between the groups, it supports prior work showing that subtle cognitive changes can occur in the absence of or independent of Aβ [38, 50].

A strength of our current study is that actuarial neuropsychological criteria for MCI were used to exclude MCI participants [32, 33]. This approach to classifying MCI likely led to a more accurate pool of CU individuals from which to classify SMC and Obj-SCD given prior work showing that this approach leads to a more reliable MCI group [31, 32]. The neuropsychological measures used to define Obj-SCD require more training and time to administer than the single CCI questionnaire. The inclusion of multiple neuropsychological scores in the Obj-SCD criteria likely yielded a more reliable characterization of one’s cognitive performance than the single measure of subjective memory changes. Future work may wish to examine whether an actuarial approach for SMC classification (i.e., that requires rating above a threshold on multiple SMC measures) improves reliability compared to a single cut score on one measure or composite score. Although it is possible that the SMC group is capturing an “earlier” pre-MCI phase than the Obj-SCD group, and therefore does not have tau that has progressed to Braak stage III/IV, there were also no differences in Aβ burden between SMC− and SMC+ groups. Furthermore, there is evidence that cognitively unimpaired but Aβ-positive participants exhibit a widespread distribution of tau relative to Aβ-negative participants that extends beyond the medial temporal lobe, including to the lateral temporal lobe and parietal regions [36]. Therefore, it is likely that if the SMC group were accurately discriminating people on an AD trajectory, they would have at least a pattern for higher tau levels in limbic regions captured by Braak stages III/IV.

### Limitations

A significant limitation of the current study is that the sample is not representative of the population or those most at-risk for AD given that it is predominately white, highly educated, and generally very healthy. Further work is needed to examine these relationships in more diverse cohorts, particularly since the Obj-SCD classification may have reduced utility for people for whom the neuropsychological measures were not originally validated given that many tests were initially developed and tested on white, educated volunteers. Another limitation of the study is the small number of participants with Obj-SCD but not SMC. While this group had the greatest tau burden, the results should be replicated in a larger sample. Longitudinal analyses comparing cognitive and biomarker trajectories of SMC and Obj-SCD are needed to make definitive conclusions related to whether one measure is more sensitive and reliable to capturing an earlier pre-MCI phase than the other, or if they are measuring different aspects of the pre-MCI phase.

## Conclusions

In summary, our study shows that Obj-SCD, but not SMC, is associated with higher tau PET levels. There was very little classification agreement between Obj-SCD and SMC, and the addition of SMC to the Obj-SCD method did not improve the Obj-SCD association with the tau burden. These findings add to the existing evidence that the Obj-SCD classification is associated with AD biomarkers and faster cognitive decline in ADNI participants, but further work is needed to validate this classification approach in more representative/diverse cohorts before it can be applied in a clinical setting or as a tool for clinical trial screening.

## Data Availability

Alzheimer’s Disease Neuroimaging Initiative data are available at http://adni.loni.usc.edu/data-samples/access-data/ to anyone approved to access ADNI data. Derived data used during the current study are available from the corresponding author upon reasonable request.

## Abbreviations

Aβ: β-Amyloid
AD: Alzheimer’s disease
ADNI: Alzheimer’s Disease Neuroimaging Initiative
APOE: Apolipoprotein E
AVLT: Rey Auditory Verbal Learning Test
CCI: Cognitive Change Index
CU: Cognitively unimpaired
ECog: Everyday Cognition
GDS: Geriatric Depression Scale
Obj-SCD: Objective subtle cognitive decline
PACC: Preclinical Alzheimer’s Cognitive Composite
PET: Positron emission tomography
ROI: Region of interest
SMC: Subjective memory complaint
SUVR: Standardized uptake variable ratio
T: Tau
